# Supplementary material for: NAC Transcription Factor GmNAC12 Improved Drought Stress Tolerance in Soybean
Source: Int J Mol Sci. 2022 Oct 10;23(19):12029. doi: 10.3390/ijms231912029 (PMC9570484; doi:10.3390/ijms231912029)
Supplement: Supplementary file 1 [file ijms-23-12029-s001.zip › Supplementary Figure.pdf]

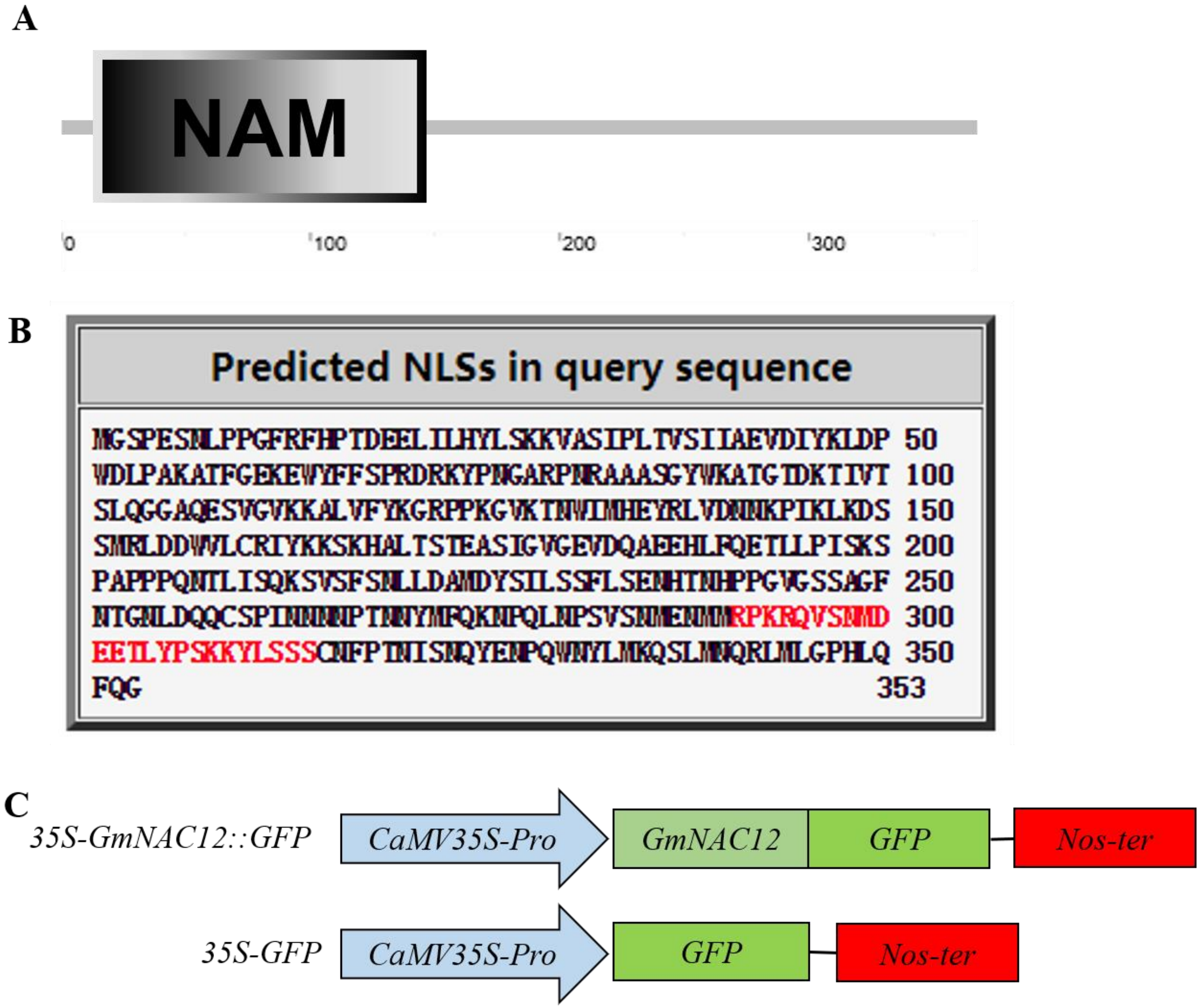

Figure S1. GmNAC12 contains a NAM domain and two NLSs. (**A**) Amino acid sequence analysis of GmNAC12. (**B**) Prediction the nuclear localization signals of GmNAC12 by cNLS Mapper. (**C**) Construction of GmNAC12 subcellular localization vector.
