# Supplementary material for: NAC Transcription Factor GmNAC12 Improved Drought Stress Tolerance in Soybean
Source: Int J Mol Sci. 2022 Oct 10;23(19):12029. doi: 10.3390/ijms231912029 (PMC9570484; doi:10.3390/ijms231912029)
Supplement: Supplementary file 1 [file ijms-23-12029-s001.zip › Supplementary Table.pdf]

Table S1

Primers used to isolate the *GmNAC12*

Gene	Sequence of primer pair (5’-3’)
GmNAC12	TTTCCTTTCAACCTTATCAAATTAA/GGTGTGCTCAGCTTCTGAATAT

Primers used for quantitative real-time PCR

Gene	Sequence of primer pairs (5’-3’)
GmNAC12	TCCAAGAGACCCTTTTGCCA/TGGTGGGTGGTTGGTATGAT
GmActin11	GGTGGTTCTATCTTGGCATC/CTTTCGCTTCAATAACCCTA

Primers used to construct vector for subcellular localization

Gene	Sequence of primer pairs (5’-3’)
GmNAC12	CGGGGTACCATGGGAAGCCCAGAATCCAATTTG/<br>TTCCGCGGCCGCTATGGCCGACGTCGACTCCTTGAAATTGAAGATGAGGA

Primers used to construct vector for Crispr/Cas9 target and test

Gene	Sequence of primer pairs (5’-3’)
GmNAC12-KO-20bp	ATCAAGCTCAAAGACTCCTC
GmNAC12-KO-test	GTCCTAGAGACCGAAAGTACCC/CCACATGTTGATTGCATATGGC

Primers used to construct vector for plant overexpression

Gene	Sequence of primer pairs (5’-3’)
GmNAC12-OE	GCTCTAGAATGGGAAGCCCAGAATCCAATTTG/<br>GGACTAGTTTATCCTTGAAATTGAAGATGAGGA

Primers used to construct vector for yeast two-hybrid

Gene	Sequence of primer pairs (5’-3’)
GmNAC12	CATGGAGGCCGAATTCATGGGAAGCCCAGAATCCA/<br>GGATCCCCGGGAATTCTCCTTGAAATTGAAGATGAGGACCA

Table S2. GO annotations of GmNAC12 candidate interaction proteins

ID	term	category	gene
GO:0006412	translation	biological process	Glyma.02G047800; Glyma.14G030000; Glyma.03G197600; Glyma.10G221600; Glyma.17G098600; Glyma.06G192500; Glyma.03G241100; Glyma.04G139200; Glyma.02G220000; Glyma.01G140100; Glyma.07G198600
GO:0006635	fatty acid beta-oxidation	biological process	Glyma.07G239200; Glyma.15G027800; Glyma.11G035200
GO:0042742	defense response to bacterium	biological process	Glyma.15G126400; Glyma.19G245500; Glyma.07G015800; Glyma.19G245600; Glyma.02G062500; Glyma.17G057100; Glyma.19G245400
GO:0051607	defense response to virus	biological process	Glyma.05G105800; Glyma.03G201100; Glyma.19G198900
GO:0018298	protein-chromophore linkage	biological process	Glyma.14G008000; Glyma.05G128000; Glyma.08G082900
GO:0042744	hydrogen peroxide catabolic process	biological process	Glyma.12G073100; Glyma.05G103600; Glyma.14G201700; Glyma.20G001400
GO:0009834	plant-type secondary cell wall biogenesis	biological process	Glyma.19G180700; Glyma.12G069300; Glyma.05G161900
GO:0050832	defense response to fungus	biological process	Glyma.15G126400; Glyma.19G245500; Glyma.19G245600; Glyma.12G240400; Glyma.19G245400
GO:0007264	small GTPase mediated signal transduction	biological process	Glyma.05G105800; Glyma.05G176300; Glyma.13G333500
GO:0009408	response to heat	biological process	Glyma.16G101900; Glyma.07G140400; Glyma.11G078100; Glyma.02G062500
GO:0022627	cytosolic small ribosomal subunit	cellular component	Glyma.02G047800; Glyma.10G221600; Glyma.17G098600; Glyma.06G192500; Glyma.11G021700
GO:0005840	ribosome	cellular component	Glyma.14G030000; Glyma.03G197600; Glyma.10G221600; Glyma.03G241100; Glyma.04G139200; Glyma.02G220000; Glyma.07G198600; Glyma.02G002800
GO:0009522	photosystem I	cellular component	Glyma.14G008000; Glyma.05G128000; Glyma.08G082900
GO:0022626	cytosolic ribosome	cellular component	Glyma.02G047800; Glyma.10G221600; Glyma.01G140100; Glyma.11G021700; Glyma.13G245300
GO:0009523	photosystem II	cellular component	Glyma.14G008000; Glyma.05G128000; Glyma.08G082900
GO:0009535	chloroplast thylakoid membrane	cellular component	Glyma.02G007700; Glyma.14G008000; Glyma.20G110400; Glyma.09G250800; Glyma.05G128000; Glyma.08G082900; Glyma.17G130100; Glyma.05G048000; Glyma.19G007800
GO:0009506	plasmodesma	cellular component	Glyma.05G105800; Glyma.03G201100; Glyma.13G043900; Glyma.10G221600; Glyma.08G071300; Glyma.17G098600; Glyma.06G192500; Glyma.01G140100; Glyma.11G035200; Glyma.19G198900; Glyma.05G070300
GO:0016607	nuclear speck	cellular component	Glyma.03G188600; Glyma.18G195500; Glyma.19G191100
GO:0016020	membrane	cellular component	Glyma.13G285300; Glyma.10G221600; Glyma.11G078100; Glyma.18G225300; Glyma.07G015800; Glyma.17G098600; Glyma.06G192500; Glyma.01G140100; Glyma.14G048800; Glyma.05G070300; Glyma.11G021700; Glyma.07G039800
GO:0005622	intracellular	cellular component	Glyma.17G186600; Glyma.19G245600; Glyma.02G062500; Glyma.05G176300
GO:0003735	structural constituent of ribosome	molecular function	Glyma.02G047800; Glyma.14G030000; Glyma.03G197600; Glyma.10G221600; Glyma.17G098600; Glyma.06G192500; Glyma.03G241100; Glyma.04G139200; Glyma.02G220000; Glyma.01G140100; Glyma.07G198600; Glyma.11G021700; Glyma.13G245300

GO:0008061	chitin binding	molecular function	Glyma.19G245500; Glyma.11G124500; Glyma.19G245400
GO:0016168	chlorophyll binding	molecular function	Glyma.14G008000; Glyma.05G128000; Glyma.08G082900
GO:0019843	rRNA binding	molecular function	Glyma.02G047800; Glyma.10G221600; Glyma.17G098600; Glyma.06G192500; Glyma.13G151300
GO:0004842	ubiquitin-protein transferase activity	molecular function	Glyma.15G126400; Glyma.18G195500; Glyma.08G153200
GO:0003729	mRNA binding	molecular function	Glyma.02G047800; Glyma.17G240400; Glyma.10G221600; Glyma.01G140100; Glyma.11G021700
GO:0050660	flavin adenine dinucleotide binding	molecular function	Glyma.13G199600; Glyma.11G035200; Glyma.05G124900
GO:0004601	peroxidase activity	molecular function	Glyma.05G103600; Glyma.14G201700; Glyma.20G001400
GO:0042802	identical protein binding	molecular function	Glyma.03G015500; Glyma.13G199600; Glyma.07G257500
GO:0020037	heme binding	molecular function	Glyma.13G285300; Glyma.12G073100; Glyma.05G103600; Glyma.14G201700; Glyma.07G039800; Glyma.20G001400

Table S3. KEGG pathway of GmNAC12 candidate interaction proteins

ID	term	gene
ko03010	ribosome	Glyma.03G197600; Glyma.01G140100; Glyma.13G245300; Glyma.03G241100; Glyma.04G139200; Glyma.17G098600; Glyma.02G220000; Glyma.07G198600; Glyma.06G192500; Glyma.11G021700; Glyma.02G047800; Glyma.02G002800; Glyma.10G221600
ko00196	photosynthesis - antenna proteins	Glyma.05G128000; Glyma.08G082900; Glyma.14G008000
ko00983	drug metabolism - other enzymes	Glyma.05G222300; Glyma.10G125100; Glyma.07G140400; Glyma.03G109500
ko04371	apelin signaling pathway	Glyma.14G040600; Glyma.07G198600; Glyma.13G060400
ko00195	photosynthesis	Glyma.09G250800; Glyma.17G130100; Glyma.05G048000
ko04144	endocytosis	Glyma.13G333500; Glyma.14G018200; Glyma.11G099100; Glyma.16G101900; Glyma.14G048800; Glyma.05G176300
ko00940	phenylpropanoid biosynthesis	Glyma.05G124900; Glyma.20G001400; Glyma.15G003600; Glyma.05G103600; Glyma.14G201700; Glyma.08G220200
ko00480	glutathione metabolism	Glyma.19G226700; Glyma.07G140400; Glyma.12G073100
ko03018	RNA degradation	Glyma.07G184300; Glyma.17G240400; Glyma.16G042600
ko00240	pyrimidine metabolism	Glyma.05G222300; Glyma.16G043700; Glyma.03G109500
ko00190	oxidative phosphorylation	Glyma.17G130100; Glyma.18G244300; Glyma.05G048000
ko00500	starch and sucrose metabolism	Glyma.13G114000; Glyma.13G088300; Glyma.07G172000
ko03013	RNA transport	Glyma.17G186600; Glyma.17G240400; Glyma.19G252400
ko04016	MAPK signaling pathway - plant	Glyma.14G040600; Glyma.12G073000; Glyma.03G109500
ko00230	purine metabolism	Glyma.16G043700; Glyma.10G125100; Glyma.03G109500
